# Cardiovascular Function of Modern Pigs Does not Comply with Allometric Scaling Laws

**DOI:** 10.1038/s41598-017-18775-z

**Published:** 2018-01-15

**Authors:** Gerard J. van Essen, Maaike te Lintel Hekkert, Oana Sorop, Ilkka Heinonen, Jolanda van der Velden, Daphne Merkus, Dirk J. Duncker

**Affiliations:** 1 Division of Experimental Cardiology, Department of Cardiology, Thoraxcenter, Cardiovascular Research School COEUR, Erasmus MC, University Medical Center Rotterdam, Rotterdam, The Netherlands; 2 Turku PET Centre, and Department of Clinical Physiology and Nuclear Medicine, University of Turku, Turku, Finland; 3 Department of Physiology, Amsterdam Cardiovascular Sciences, VU University Medical Center, Amsterdam, The Netherlands; 4 Netherlands Heart Institute, Utrecht, The Netherlands

## Abstract

Growing concerns have been expressed regarding cardiovascular performance in modern farm pigs, which has been proposed as a critical factor contributing to the reduced adaptability of modern pigs to stress. Here we tested the hypothesis that cardiac dimensions and pump function in modern heavy farm pigs are disproportionally low for their body weight, and investigated potential underlying mechanisms. The results from the present study indeed demonstrate disproportionally low values for stroke volume and cardiac output in pigs with bodyweights over 150 kg. Importantly, these low values were not the result of impaired left ventricular (LV) systolic contractile function, but were due to a disproportionally small LV end-diastolic volume. The latter was associated with changes in determinants of LV passive stiffness, including (*i*) an increase in LV myocardial collagen, (*ii*) a shift from the compliant N2BA titin isoform towards the stiff N2B, and (*iii*) a marked elevation of aortic blood pressure. Taken together, these results demonstrate reduced pumping capacity of the hearts of heavy modern pigs, due to structural abnormalities in the LV myocardium.

## Introduction

Modern pigs originate from wild boar^1^ with domestication starting in the Near East as early as 9000 years B.C.^2^. Domestication of the wild boar has, particularly during the last century, resulted in marked increases in litter size, body weight (BW) and muscularity^3,4^, which has inadvertently led to alterations of the cardiovascular system, including a lower blood volume and haemoglobin, as well as a lower relative heart weight (HW) and cardiac output (CO)^5,6^. These changes, which raised concerns with respect to function and adaptability to stress in modern farm pigs^5,7^, have been ascribed to a loss of proportionality of HW and CO with BW in modern fast growing pigs^5–7^. Early studies assessed proportionality by simply dividing HW and CO by BW^5,6^. However, more recent studies have shown that in mammals, HW and CO scales with BW to the power of 0.75, and stroke volume (SV) scales with BW to the power of 1.00^8–10^. The observed scaling is typically a simple power law: Y = a·BW^b^, where Y is the observed variable, for example CO, “a” is a constant and the exponent b almost invariably approximates a multiple of 0.25.

Previously we tested the scaling hypothesis for CO and SV in modern growing pigs and in adult sows at the end of their gestation, and found that, while CO and SV of pigs up to 75 kg scaled proportionally with BW^11^, adult sows demonstrated a disproportionally low CO and SV^12^. In our previous studies, we included two sets of pigs that had different genetic backgrounds, underwent different anesthesia regimens and were studied in different laboratory settings. Moreover, in our previous study we did not further investigate the mechanisms underlying the disproportionally low levels of SV in adult pigs. Consequently, in the present study we investigated the proportionality of the cardiovascular system over a wide range of BW (22–216 kg) in pigs of a single (female) sex, with a similar genetic background, undergoing invasive haemodynamic studies in a single laboratory using a uniform anesthesia regimen. Importantly, in the present study we also explored the mechanisms underlying the disproportionally low SV by assessing left ventricular dimensions as well as LV systolic and diastolic function, and by performing myocardial tissue analysis.

## Results

### Anatomical and physiological results

Table 1 shows the anatomical and physiological data for the three weight categories. Individual data can be found as Supplementary Data S1. HW and LVW, as well as LV dimensions increased significantly with increasing BWs (all P < 0.001). CO also increased with increasing BW, which was the result of an increase in SV (both P < 0.001), as heart rates were not significantly different between the three weight groups. Mean pulmonary artery pressure and pulmonary capillary wedge pressure also did not differ significantly between the three weight categories, but mean arterial pressure and LV systolic pressure were markedly elevated in the highest weight category (P < 0.001 and P = 0.002 vs < 75 kg-group respectively). LV systolic function (reflected by LVdP/dt_P=40_ and both 2D and 3D ejection fractions), was not different between the weight groups. In contrast, although LV end-diastolic pressures were not different between weight groups, the time constant of relaxation was significantly increased in the highest weight category (P = 0.026 vs < 75 kg-group), indicating slowed LV relaxation. There were no differences between the BW categories in arterial blood pH, PCO_2_ or PO_2_.

**Table 1:** Anatomical and physiological data.

	<75 kg	n	75–150 kg	n	>150 kg	n	ANOVA
**Anatomical data**							
Body weight (kg)	46 ± 5	10	106 ± 4*	13	182 ± 8*^,†^	8	<0.001
Heart weight (g)	147 ± 10	4	367 ± 26*	8	612 ± 33*^,†^	7	<0.001
LV weight (g)	112 ± 7	9	233 ± 14*	9	381 ± 17*^,†^	8	<0.001
LV end diastolic lumen volume (mL)	81 ± 12	6	154 ± 20*	8	283 ± 19*^,†^	7	<0.001
LV end diastolic lumen area (mm^2^)	1510 ± 90	6	2600 ± 310*	3	2810 ± 160*	6	<0.001
**Haemodynamics**							
Heart rate (bpm)	96 ± 7	10	85 ± 4	13	80 ± 8	8	0.164
Mean arterial pressure (mmHg)	84 ± 8	10	109 ± 4*	13	138 ± 12*^,†^	8	<0.001
Mean pulmonary artery pressure (mmHg)	22 ± 2	9	26 ± 2	13	27 ± 3	7	0.425
Pulmonary capillary wedge pressure (mmHg)	9 ± 1	9	12 ± 1	12	12 ± 1	6	0.112
Cardiac output (L·min^−1^)	4.3 ± 0.4	10	6.0 ± 0.4*	12	8.5 ± 0.6*^,†^	8	<0.001
Stroke volume (mL·beat^−1^)	45 ± 4	10	75 ± 9*	12	115 ± 16*^,†^	8	<0.001
**LV function**							
LV systolic pressure (mmHg)	109 ± 4	9	122 ± 5	13	154 ± 14*^,†^	7	0.002
LV dP/dt_P=40_ (mmHg·s^−1^)	1390 ± 140	9	1180 ± 80	13	1310 ± 50	7	0.316
2-D LV ejection fraction (%)	39 ± 3	6	44 ± 9	3	45 ± 3	6	0.470
3-D LV ejection fraction (%)	61 ± 4	6	60 ± 7	8	57 ± 5	7	0.881
tau (ms)	48 ± 2	9	54 ± 3	13	64 ± 6*	7	0.034
LV end diastolic pressure (mmHg)	12 ± 2	9	16 ± 2	13	13 ± 2	7	0.365
**Arterial blood gasses**							
pH	7.44 ± 0.02	7	7.45 ± 0.02	9	7.44 ± 0.01	8	0.863
PCO_2_ (mmHg)	41 ± 1	7	40 ± 2	9	38 ± 2	8	0.437
PO_2_ (mmHg)	140 ± 8	7	150 ± 7	9	148 ± 10	8	0.693

LV = left ventricular; LV dP/dt_P=40_ = rate of rise in LV pressure at 40 mmHg; tau = time constant of LV pressure decay during early diastole; pH = −log (hydrogen ion concentration); PCO_2_ = partial carbon dioxide pressure; PO_2_ = partial oxygen pressure; LV end diastolic lumen area and 2-D LV Ejection fraction measured with open chest; Data are mean ± s.e.m.; *P < 0.05 vs < 75 kg-group; ^†^P < 0.05 vs 75–150 kg-group (by One-Way ANOVA followed by SNK post-hoc testing).

### Scaling of Cardiac Geometry and Function to Body Weight

**Figure 1:**
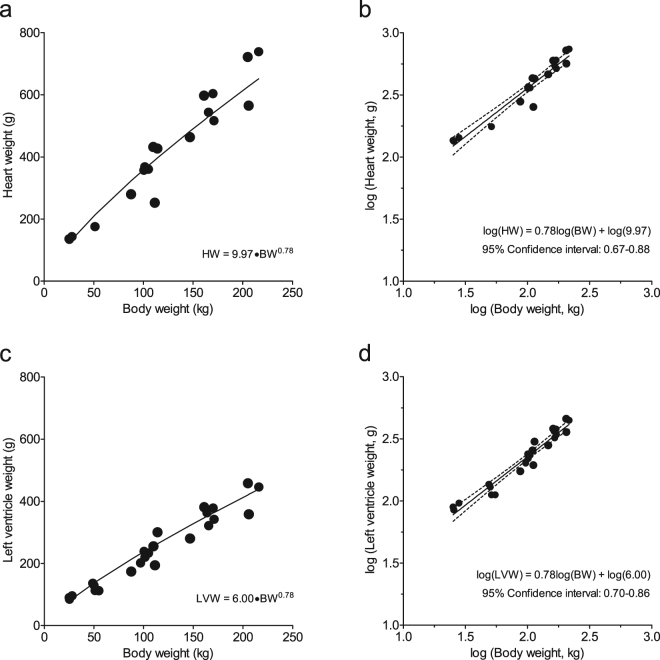
Relations between heart weight and body weight (panels a and b) and left ventricular weight and body weight (panels c and d). Absolute values of heart Heart weight (HW) and left ventricle weight (LVW), respectively, plotted to body weight (panels a and c). To linearize the formulas HW = 9.97 × BW^0.78^ and LVW = 6.00×BW^0.78^, the common logarithm was taken: log(HW or LVW) = log(a) + b.log(BW), with the predicted value of exponent b of 0.75 falling well within the 95% confidence limits (panels b and d). This indicates that HW and LVW scale proportionally with body weight according to the natural scaling laws.

**Figure 2:**
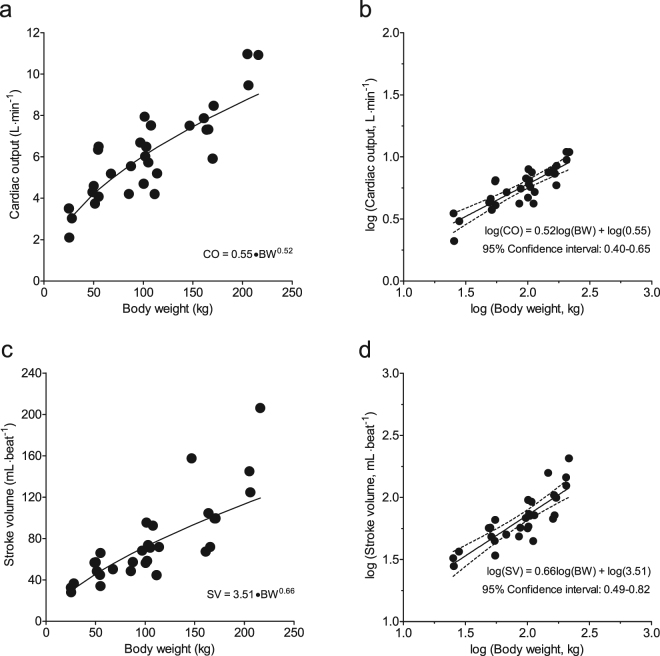
Relations between cardiac output and body weight (panels a and b) and stroke volume and body weight (panels c and d). Absolute values of cardiac output (CO) and stroke volume (SV), respectively, plotted to body weight (panels a and c). To linearize the formulas CO = 0.55 × BW^0.52^ and SV = 3.51·BW^0.66^, the common logarithm was taken: log(CO or SV) = log(a) + b.log(BW), with the predicted exponents b of 0.75 for CO, and 1.00 for SV falling outside the 95% confidence interval (panels b and d). This indicates that CO and SV failed to scale proportionally with body weight according to the natural scaling laws.

**Figure 3:**
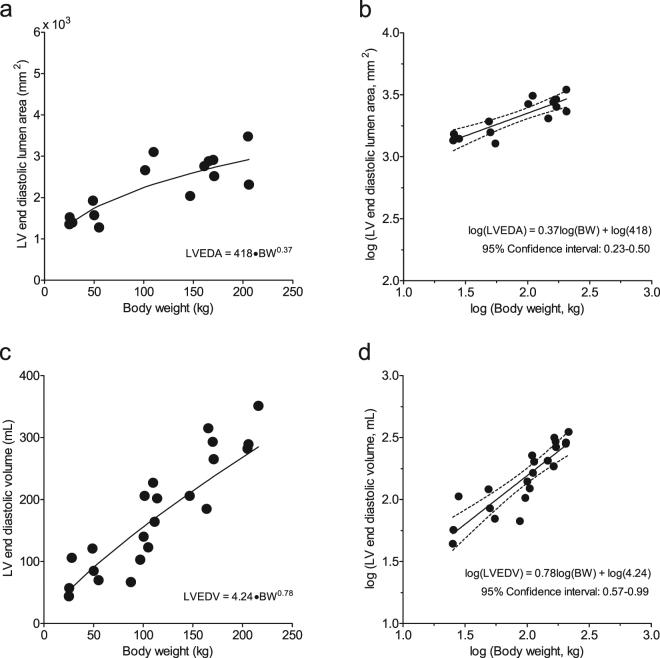
Relations between LV end-diastolic lumen area and body weight (panels b and d) and LV end-diastolic lumen volume and body weight (panels c and d). Absolute levels of left ventricular end-diastolic lumen area (LVEDA) and LV end-diastolic lumen volume (LVEDV), respectively, plotted to body weight (panels a and c). To linearize the formulas LVEDA = 418·BW^0.37^ and LVEDV = 4.24·BW^0.78^, the common logarithm was taken: log(LVEDA or LVEDV) = log(a) + b.log(BW), with the predicted exponents b of 0.67 for LVEDA, and 1.00 for LVEDV falling outside the 95% confidence interval (panels b and d). This indicates that LVEDA and LVEDV failed to scale proportionally with body weight according to the natural scaling laws.

To test the hypothesis that cardiac weight, dimensions and function of modern growing pigs are proportional to BW, as predicted by the quarter scaling laws, we plotted various parameters as a function of BW. HW and LVW increased with BW according to the formulas HW = 9.97 × BW^0.78^ and LVW = 6.00 × BW^0.78^ (Fig. 1), with the predicted value of exponent b of 0.75^9,13^ falling well within the 95% confidence limits (Table 2), indicating that both HW and LVW scale proportionally with BW according to the natural scaling laws. In contrast, CO and SV increased with BW described by the formulas: CO = 0.55 × BW^0.52^ and SV = 3.51·BW^0.66^ (Fig. 2), with the predicted exponents b of 0.75 for CO, and 1.00 for SV^9,13^ falling outside the 95% confidence intervals (Table 2), indicating that CO and SV failed to follow natural scaling laws. These observations were paralleled by a similar disproportionality of LV dimensions. Thus, LVEDA (measured with echocardiography) and LVEDV (measured with the conductance catheter), increased with BW according to the formula LVEDA = 418·BW^0.37^ and LVEDV = 4.24·BW^0.78^ (Fig. 3), with the confidence limits failing to encompass the predicted exponents b of 0.67 for LVEDA^14^ and 1.00 for LVEDV^8,15^ (Table 2).

**Table 2:** Confidence intervals of coefficient b.

	Lower	Mean	Upper	Predicted
Heart weight	0.67	0.78	0.88	0.75
LV weight	0.70	0.78	0.86	0.75
Cardiac output^#^	0.40	0.52	0.65	0.75
Stroke volume^#^	0.49	0.66	0.82	1.00
LV EDA^#^	0.23	0.37	0.50	0.67
LV EDV^#^	0.57	0.78	0.99	1.00

Shown are the 95% confidence intervals for coefficient b for anatomical and haemodynamic parameters of the pig heart; LV = left ventricle; EDA = end diastolic lumen area; EDV = end diastolic lumen volume, ^#^the predicted value is outside the confidence interval.

### Myocardial Collagen and Titin Composition

**Figure 4:**
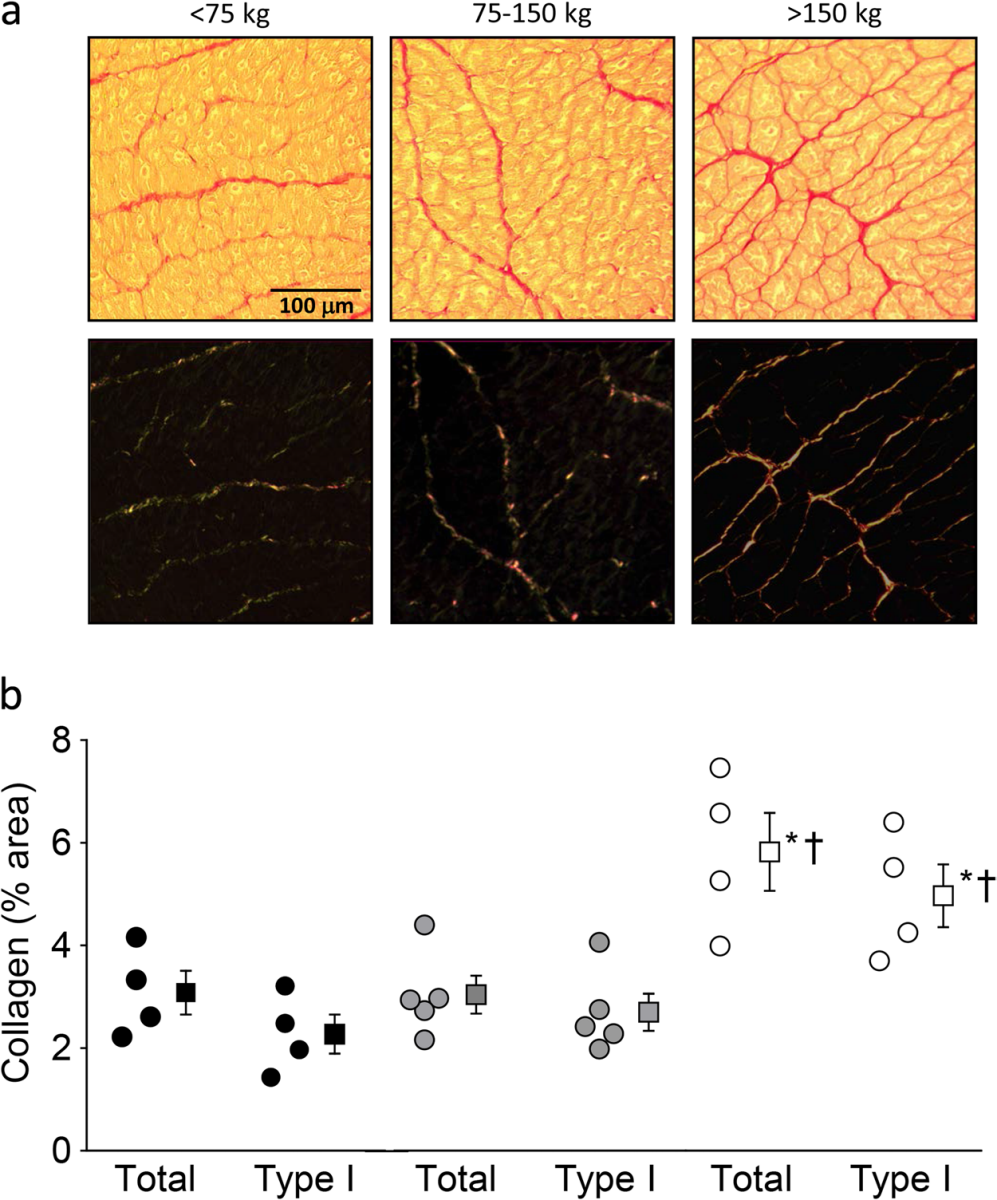
Myocardial collagen content in an animal in each of the three weight categories. (**a**) Collagen staining (picrosirius red) of LV myocardial sections. Upper panels show slides using light microscopy, while lower panels show same tissue slides using a linear polarization filter. (**b**) Bar graph showing amount of collagen as percentage of myocardium area. Total collagen and collagen type I (thick fibers) are increased in the highest weight category. Measured in a subset within each group (n = 4, n = 5 and n = 4 respectively for each group); Data are presented as individual animal data points and as mean ± s.e.m.; *P < 0.05 vs < 75 kg-group; ^†^P < 0.05 vs 75–150 kg-group (by One-Way ANOVA followed by SNK post-hoc testing).

**Figure 5:**
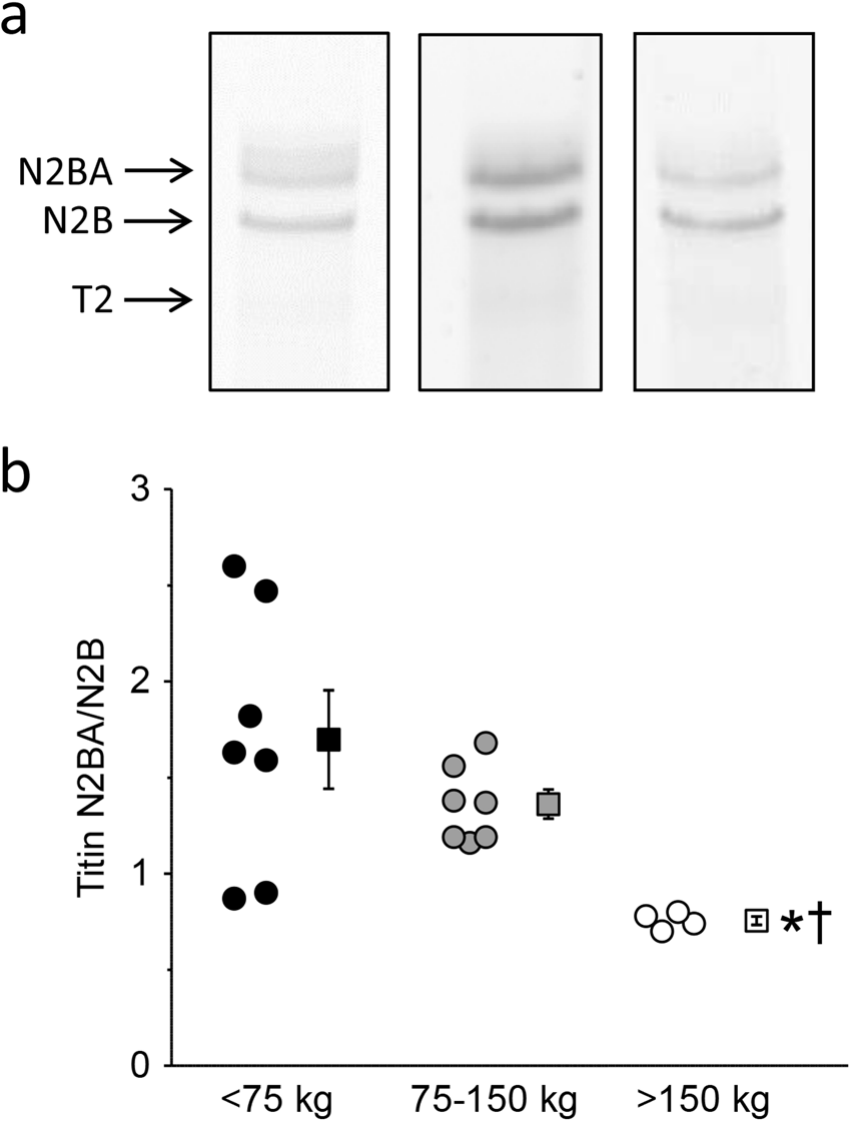
Titin isoform content in an animal in each of the three weight categories. (**a**) Gels showing titin isoform staining using SYPRO Ruby protein staining. Each example is from a different gel and gels are cropped. (Full length gels are included in Supplementary Information S4). The grayscale of the 75–150 kg-group gel is adjusted to generate equal appearance of the three gels. (**b**) Bar graph showing titin ratio (N2BA/N2B isoform). Ratio is decreased in the highest weight category, indicating a shift from the compliant N2BA titin isoform towards the stiff N2B titin isoform. Measured in a subset within each group (n = 7, n = 7 and n = 4, respectively, for each group); Data are presented as individual animal data points and as mean ± s.e.m.; *P < 0.05 vs < 75 kg-group; ^†^P < 0.05 vs 75–150 kg-group (by One-Way ANOVA followed by SNK post-hoc testing).

Histological analysis of LV tissue demonstrated an increase in total collagen and collagen type I (thick fibers) in the highest BW category (Fig. 4; Individual data can be found as Supplementary Data S2), suggestive of increased fibrosis in this group. Furthermore, in small LV myocardial tissue samples, a shift from the compliant N2BA titin isoform towards the stiff N2B titin isoform was evident in the highest BW category animals, as indicated by a lower N2BA/N2B ratio compared to the low BW category pigs (Fig. 5; Individual data can be found as Supplementary Data S3; Full length gels can be found as Supplementary Information S4). T2, the degradation product of titin, did not differ among groups and, in accordance with titin analyses in other species^16^, was very low.

## Discussion

The present study was performed in modern farm pigs, covering a wide range of BWs, to test the hypothesis that cardiac weights, dimensions and function obey quarter power scaling laws. The main findings were that: (*i*) HW and LVW increase commensurately with BW according to the quarter power scaling laws; (*ii*) CO and SV failed to increase proportionally to BW, which (*iii*) was not due to impaired LV systolic function, but rather (*iv*) appeared due to a similar lack of proportional increase in LV dimensions, as measured with echocardiography and conductance catheter; (*v*) haemodynamic analysis revealed marked increases in arterial blood pressure, while analysis of myocardial tissue revealed increases in interstitial collagen content and in the stiff N2B titin isoform of the myocytes in animals with the highest BW. The implications of these findings will be discussed below.

In 1932, Max Kleiber published a seminal paper, showing that metabolic rates among mammals varied with three–quarter power of BW^17,18^. Since then, many scientists have reported similar allometric scaling phenomena in mammals^19^. Allometric scaling is defined as a simple power law relation between BW and various anatomical and physiological variables. West and Brown have proposed that metabolic rate plays a key role in determining the scale of biological phenomena and that the existence of a fourth spatial dimension in mammals explains why allometric scaling is quarter-power scaling^10,13^. These biological quarter-power scaling laws indicate that many biological variables scale to BW to the power of b and the exponent b almost invariably approximates a simple multiple of ¼. Quarter power scaling of cardiovascular parameters is widely used in cardiovascular medicine, for example in pediatric clinical practice^20^ and in veterinary practice^14,21^.

Using the quarter-power scaling laws we have investigated the cardiovascular proportionality of modern pigs^11,12^. In those previous studies we found that, while in growing and in adult pigs the HW was proportional to BW, SV and CO were proportional only in pigs up to 75 kg^11^, but were disproportionally low in sows at the end of their gestation^12^. However, in those studies, pigs were included that had different genetic backgrounds, were either male or female, were subjected to different anesthesia regimens and were studied in different laboratory settings. Consequently, in the present study we included only pigs of a single (female) sex, with similar genetic background, over a wide range of BWs, subjected to studies in a single laboratory using a uniform anesthesia regimen. The present study confirms our previous observations of disproportionally low values of SV and CO, with remarkably similar values of the exponent b for HW, SV and CO^11^. In addition, the present study explored several mechanisms that could contribute to the low values of SV.

A low SV can be caused by a decrease in systolic function. However, indices of LV systolic function, including LVdP/dt_P=40_, and 2-D and 3-D ejection fractions were maintained suggesting that LV systolic function was well maintained in animals in the highest weight group. Importantly, a reduction in SV can also result from perturbations in diastolic function. Indeed, using two independent techniques to assess LV volume (conductance catheter) and LV short axis lumen area (echocardiography), we observed disproportionally low values of LVEDV and LVEDA. The exact mechanism for the disproportionally small end-diastolic LV dimensions in adult animals remains to be established but, based on the present study, several potential mechanisms could be proposed. First, the time constant of relaxation (tau) was increased indicating slowing of LV relaxation and impeding filling in the early rapid LV filling phase. Moreover, LV myocardial interstitial levels of total collagen and the thick fiber collagen type I were significantly higher in adult pigs, which both act to increase passive LV stiffness^22^. In addition, we observed a shift from the compliant titin isoform N2BA to the stiff titin isoform N2B in LV tissue samples, reflected by a lower ratio of N2BA/N2B in the highest BW category (>150 kg), which also acts to increase passive LV stiffness, and hence impair LV diastolic filling^23^.

Furthermore, we observed 40–60% higher LV systolic and mean arterial pressures in the highest BW category. Chronic LV pressure overload likely contributed to the increase in interstitial collagen levels and the impaired LV relaxation that we observed in the present study^24^. However, the 41% increase in LV systolic pressure would also be expected to result in LV hypertrophy. Yet, LV weight did not appear to be disproportionally increased according to the quarter-power scaling law. Although an explanation for this unexpected finding is not readily found, it could be speculated that LV weight in fact scaled proportionally to BW just because of a hypertrophic response that may have acted to mask a disproportionally low LV weight in the heavy pigs. Future studies, using vasodilator drugs to reduce blood pressure, are required to test this hypothesis and investigate the scaling of LV weight to BW in adult pigs in the presence of a normal blood pressure.

The present study provides further evidence for the concept that LV dimensions and function do not scale with BW in modern pigs according to allometric scaling laws, and shows that this is associated with pathological changes within the LV myocardium. The question then arises as to how domestication has led to structural and functional changes in the heart of modern pigs. Von Engelhardt^5^ suggested that the small heart in relation to BW appears to be the result of selective breeding for growth rate, so that cardiac growth cannot keep up with body growth. Huisman^6^ proposed that the relative low HW of modern pigs is the result of selective breeding and efficient diets. However, these suggestions were contradicted by studies that found that selection on reduction of back fat thickness and an increase of growth rate during eight generations in Yorkshire pigs resulted in a significant increase of organ weights including HW and a significant positive correlation between HW and food conversion efficiency^25^, as well as a significant negative correlation between HW and back fat thickness in pure bred and cross bred pigs^26^. The latter authors proposed that the bigger relative heart size resulting from intensive selection for leanness may not necessarily reflect a returning to a natural form, but could also be an indication of pathophysiological changes such as hypertrophic cardiomyopathy^26^. Domestication of wild boar has clearly resulted in a decrease of relative HW while rigorous selection on reproductive performance and muscle (meat production) growth in modern farm pigs, may instead have led to an increase in heart muscle weight. Interestingly, the hypertension associated pressure-overload in our heavy domestic pigs may also have contributed to a higher HW, indeed suggesting that the return of HWs towards “normal” may actually have a pathological origin and may not reflect natural form.

In addition to the influence of genetic selection, it is also likely that the sedentary lifestyle of modern pigs contributes to the disproportional scaling of LV geometry and function to BW. Indeed, in humans, lifestyle and physical (in)activity in particular is an important determinant of cardiovascular health^27^. The lifestyle of free living wild boar versus modern pigs in intensive pig industry differs markedly. Thus, wild boars are active about 12 h per day to forage food and escape from predators, and they can reach a speed of up to 40 km per hour. In contrast, modern pigs in intensive pig farming live a predominantly sedentary lifestyle, with a minimum of physical activity and a permanent loading of the gastrointestinal tract to maximize body growth. We found a significant increase of both mean arterial pressure with increasing BW and a significant increase of the LV systolic pressure in the heaviest pigs (BW > 150 kg). It is highly plausible that the sedentary lifestyle of modern pigs, with a marked decrease of physical activity during lifetime can cause physical deconditioning and a consequent increase in resting sympathetic activity^28^, which may have contributed to the elevated arterial pressure with increasing bodyweight, thereby resulting in LV pathology characterized by diastolic dysfunction and reduced LV compliance^29^. Increased systemic blood pressure is also well-known to be one of the most important risk factors for human cardiovascular disease, and is associated strongly with overweight and obesity, but the possible pathophysiological changes due to increased BW in humans are still scarce particularly at the level of the myocardium. From the cardiac perspective, the findings of the present study of disproportionally smaller LV size and increased LV wall stiffness associated with higher myocardial collagen content and titin isoform shift in adult modern farm pigs may therefore shed light on the mechanisms underlying cardiovascular risk due to lack of physical activity^30^, overweight and obesity^31^.

The implications of these findings may be several-fold. First, future studies into lifestyle influences on blood pressure and ultimately on myocardial structure and function of adult pigs are important to improve cardiovascular health of pigs in the farming industry. Second, and in conjunction with our previous observations that cardiac function scales well with body weight up to 75 Kg^11^, the present study suggests that preclinical cardiovascular studies aiming to use normal healthy pigs, should ideally include animals with body weights not exceeding 75 Kg. Finally, our present findings could be relevant for clinical medicine, by using adult pigs over 150 Kg as an animal model to study cardiovascular disease in humans.

There are several limitations in the present study that should be acknowledged. First, we used female pigs of a single mixed Yorkshire x Landrace breed in which body growth and ageing occurred in parallel. Future studies in male and female pigs of a variety of breeds, as well as adult fully grown pigs of various ages are required to investigate the influence of sex, breed and age. Second, a number of animals were included that were part of other study protocols which were run in parallel with the present study, that involved identical anesthesia regimen and experimental settings but in which not all variables were obtained, resulting in different numbers of observations within the three body weight groups.

In conclusion, the present study provides further evidence for the concept that cardiac dimensions and function do not scale with body weight in modern domestic pigs, according to allometric scaling laws. The disproportionally low stroke volume was not the result of systolic dysfunction, but the result of diastolic perturbations, likely as a result of increased myocardial interstitial collagen content and a shift towards the stiffer isoform of titin, in conjunction with marked elevations in aortic blood pressure that were particularly pronounced in pigs over 150 Kg in body weight. These findings underpin the growing concerns about intrinsic cardiovascular factors in modern domestic pigs, as well that of human inactivity and obesity, that may affect the animal’s and individual’s health. Future studies to evaluate possible health risks due to reduced function of the porcine heart are warranted.

## Methods

### Animals

All studies were performed in accordance with the Council of Europe Convention (ETS123) and the Directive (2010/63/EU) for the protection of vertebrate animals used for experimental and other scientific purposes, and with approval of the Animal Care Committee of Erasmus University Medical Center Rotterdam. Studies were performed in three groups of female Yorkshire x Landrace pigs (22–216 kg) classified according to BW: group 1 with BW below 75 kg (n = 10), group 2 with BW between 75 and 150 kg (n = 13) and group 3 with BW of more than 150 kg (n = 8). One week prior to surgery, pigs arrived for acclimatization at the central animal housing facility of Erasmus University Medical Center in Rotterdam. Twice a day, pigs were fed compound feed in accordance with their BW and had free access to drinking water. Starting twelve hours before surgery, pigs were denied access to food.

### Surgical Instrumentation and Measurements

The surgical instrumentation and measurements have been described previously^32,33^. Briefly, pigs were sedated with a cocktail of Tiletamine/Zolazepam (5 mg·kg^−1^), Xylazine (2.25 mg·kg^−1^) and atropine (0.03 mg·kg^−1^ i.m.), anesthetized with sodium pentobarbital (6 mg·kg^−1^ i.v.) and intubated for ventilation with O_2_ and N_2_ (1:3 v/v). A catheter was inserted into the right jugular vein and advanced into the superior vena cava for infusion of sodium pentobarbital (10–15 mg/kg·hr^−1^ i.v.) to maintain anesthesia. A fluid-filled catheter was also inserted into the right carotid artery for the measurement of mean arterial pressure and for sampling of arterial blood to measure arterial blood oxygen tension (PO_2_), carbon dioxide tension (PCO_2_) and pH (Acid-Base Laboratory model 800, Radiometer, Copenhagen, Denmark).

A Swan-Ganz catheter (5 corodyn TD F7, Braun, Melsungen, Germany) was inserted into the left jugular vein, via a sheath introducer, and advanced into the pulmonary artery for the measurement of mean pulmonary artery pressure and pulmonary capillary wedge pressure, and for thermodilution-based measurement of CO (Abbott Laboratories, North Chicago, Illinois, USA). A 7F conductance catheter (CD Leycom, Hengelo, The Netherlands) was inserted into the left carotid artery, via a sheath introducer, and advanced into the left ventricle (LV) for measurement of LV volume. The conductance catheter was calibrated using the thermodilution CO measurements and hypertonic saline before LV volume measurements were obtained. Subsequently, the conductance catheter was replaced by a micro-manometer-tipped catheter (SPC-370s, Millar Instruments, Houston, USA) for measurement of LV pressure (LVP) and its first derivative (LVdP/dt). LVdP/dt at a pressure of 40 mmHg (LVdP/dt_P=40_) was used as an afterload-independent index of systolic cardiac function. Heparin (10,000 I.U., i.v.) was administered before arterial blood gasses and haemodynamics were measured.

Subsequently, the chest was opened via sternotomy and the pericardial space was opened. After stabilization, ultrasound echocardiography recordings were made by placing the ultrasound probe directly onto the epicardium and obtaining short-axis recordings (Aloka SSD 4000; Aloka Company, Tokyo, Japan).

Just before sacrifice left ventricular biopsies were obtained, snap frozen and stored in liquid nitrogen until further analysis. Finally, animals were sacrificed, heart and lungs were excised and weighed. After removal of the large vessels, total HW was determined. Then, the atria and right ventricle were dissected and LVW was determined.

### Analysis of collagen and titin in cardiac tissue samples

After excision of the heart, LV anterior myocardial tissue samples were cut, fixated in 4% buffered formaldehyde and embedded in paraffin. Interstitial collagen content was quantified using picrosirius red staining. In accordance with the guidelines recently published by Schipke *et al*.^34^, slides of 4.5 μm were cut, deparaffinized and stained. Six to ten fields were examined in the subendocardial and subepicardial layer of each slide, at 20x magnification. After blood vessels, including perivascular collagen, tissue ruptures and folds had been excluded, the area to be analysed was determined and recorded using light microscopy. Subsequently, the same area was examined and analysed using a polarization filter. This method makes use of the birefringence capacity of the collagen fibres, allowing differentiation between the thick collagen fibres (red-yellow, Type I) and the thin fibres, with a lower birefringence, (greenish, Type III). Twelve to twenty fields per slide were analysed and averaged for each animal. The area occupied by collagen type I, collagen type III fibers, as well as total collagen was measured and expressed as percentage of the myocardial area. All measurements were performed using a microscopy image analysis system (Impak C, Clemex Vision Image analysis system, Clemex Technologies, Quebec, Canada). Titin isoforms in small samples of LV myocardial tissue were separated on 1% agarose gel and stained with SYPRO Ruby protein stain as described previously^35^. All samples were measured in triplicate, and the average was calculated per heart. Loading of samples was based on a test gel to determine accurate sample loading to analyse titin isoform composition within the linear detection range.

### Data and Statistical analysis

All haemodynamic and LV function data were recorded and digitized online using an eight-channel data acquisition program ATCODAS (Dataq Instruments, Akron OH) and stored on a computer for offline analysis with a program written in MatLab (The Mathworks, Natick, MA). A minimum of 15 consecutive beats were selected for analysis of the digitized haemodynamic signals.

Using a linear mixed model, the scaling coefficients of the relations between BW, as an independent variable and CO, SV, HW, left ventricular weight (LVW), left ventricular end-diastolic lumen volume (LVEDV) and left ventricular end-diastolic lumen area (LVEDA) as dependent variables were determined (GraphPad Prism 5, GraphPad Software, San Diego California, USA). To linearize the function CO = a.BW^b^ the common logarithm was taken: log(CO) = log(a) + b.log(BW). The value of b is presented with the upper and lower level of the 95% confidence interval (Table 2). The same transformation was performed for all above mentioned dependent variables (Figs 1–3, Table 2). These variables as well as all other data are also presented with pigs divided into three BW categories: <75 kg, 75–150 kg, and >150 kg (Table 1, Figs 4 and 5). To assess the statistical significance of differences between variables in the three BW categories, we used One-Way ANOVA, followed by Student-Newman-Keuls post-hoc testing when appropriate and an alpha of 0.05 (SigmaPlot11, Systat Software inc., Richmond, USA). Statistical significance was accepted when P < 0.05 (two-tailed). Data are presented as individual animal data points (Figs 1–5), and/or as mean ± s.e.m. (Table 1 and Figs 4 and 5).

### Data availability

The data that support the findings of this study are included in this published article and its supplementary online information files.

## Electronic supplementary material

Dataset 1

Dataset 2

Dataset 3

Supplemental Figure
