# Supplementary material for: Cardiovascular Function of Modern Pigs Does not Comply with Allometric Scaling Laws
Source: Sci Rep. 2018 Jan 15;8:792. doi: 10.1038/s41598-017-18775-z (PMC5768797; doi:10.1038/s41598-017-18775-z)
Supplement: Supplementary file 4 — Supplemental Figure [file 41598_2017_18775_MOESM4_ESM.pdf]

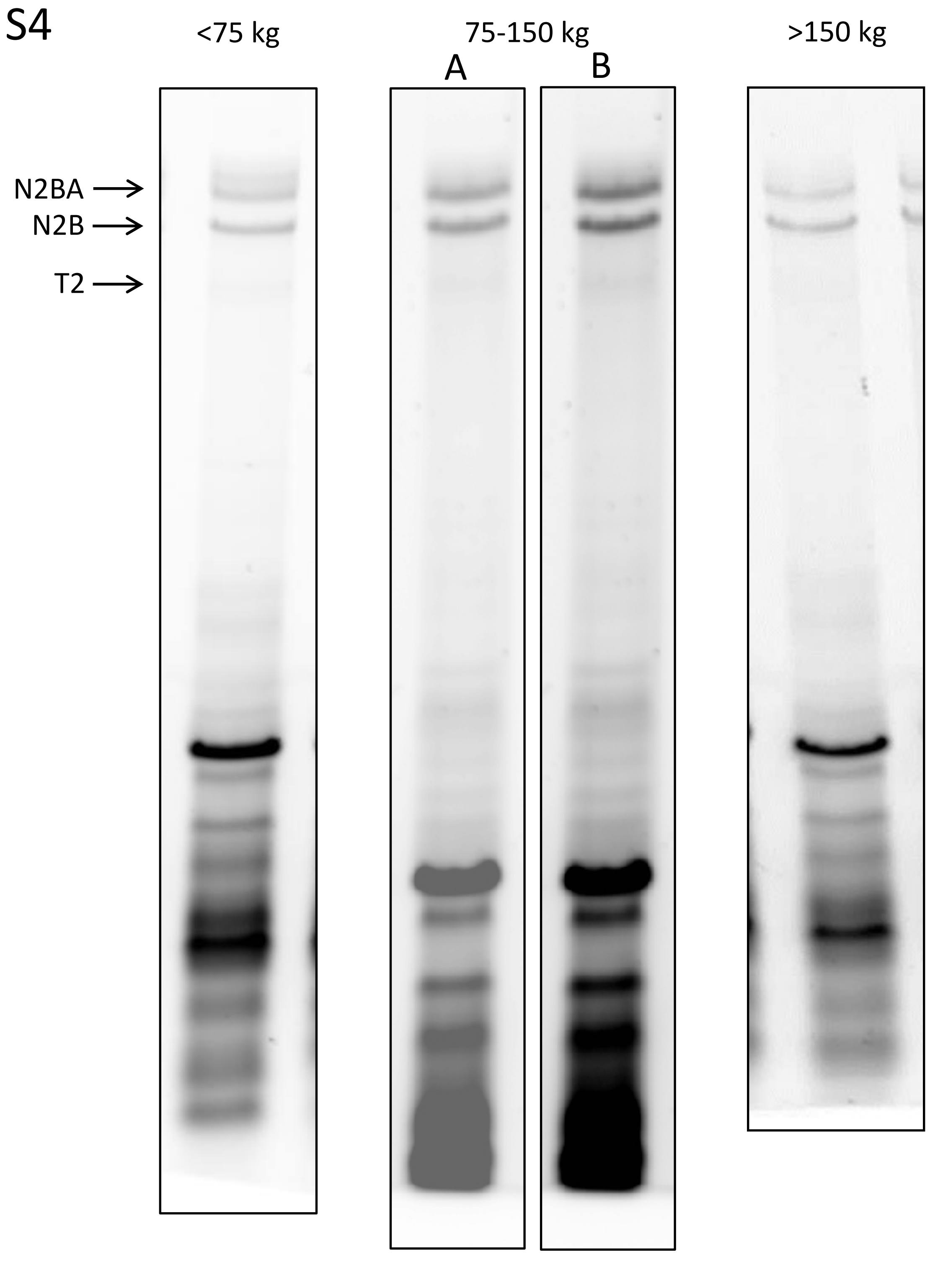


**Supplementary information S4. Full length gels from figure 5.** Each representative example is from a different gel. 75-150 kg: **A** gel as presented in manuscript, **B** original gel from A. To avoid distraction by the differences in the amount of protein between the groups, we adjusted the black/white intensity of the gel for presentation in the manuscript. We deemed this to be acceptable, since we are interested in the N2BA/N2B ratio within each gel. Importantly, for the quantitative analysis of the N2BA/N2B ratios original gels were used.
